# Reduction in Neural Performance following Recovery from Anoxic Stress Is Mimicked by AMPK Pathway Activation

**DOI:** 10.1371/journal.pone.0088570

**Published:** 2014-02-12

**Authors:** Tomas G. A. Money, Michael K. J. Sproule, Amr F. Hamour, R. Meldrum Robertson

**Affiliations:** Department of Biology, Queen's University, Kingston, Ontario, Canada; McGill University, Canada

## Abstract

Nervous systems are energetically expensive to operate and maintain. Both synaptic and action potential signalling require a significant investment to maintain ion homeostasis. We have investigated the tuning of neural performance following a brief period of anoxia in a well-characterized visual pathway in the locust, the LGMD/DCMD looming motion-sensitive circuit. We hypothesised that the energetic cost of signalling can be dynamically modified by cellular mechanisms in response to metabolic stress. We examined whether recovery from anoxia resulted in a decrease in excitability of the electrophysiological properties in the DCMD neuron. We further examined the effect of these modifications on behavioural output. We show that recovery from anoxia affects metabolic rate, flight steering behaviour, and action potential properties. The effects of anoxia on action potentials can be mimicked by activation of the AMPK metabolic pathway. We suggest this is evidence of a coordinated cellular mechanism to reduce neural energetic demand following an anoxic stress. Together, this represents a dynamically-regulated means to link the energetic demands of neural signaling with the environmental constraints faced by the whole animal.

## Introduction

The nervous systems of most animals utilize an energy budget highly disproportionate to their relative masses. High energetic costs in particular are incurred to support synaptic transmission [1] and action potential conduction [2], [3]. In response, the current form and function of neurons have been evolutionarily adapted to minimize the cost of signaling [3], [4]. Neurons with high conduction velocity and high frequency activity, nevertheless incur high energetic costs to maintain signal reliability, highlighting the trade-off that exists between decreasing energetic costs and maximizing performance. Differences in efficiency are found between different neurons [4] as well as different compartments within a neuron [5]. However, whether neural operations might be continuously tuned to balance energy consumption with performance appropriate for current or anticipated conditions is a possibility that remains almost completely unexplored.

By restricting the availability to the energetic resources of the animal, oxygen limitation itself may act as a signal to reduce the energetic demand of nervous tissue. A key difference between hypoxia-tolerant species and hypoxia-intolerant species is their relative abilities to reduce the demand that the electrogenic Na^+^/K^+^-ATPase places on a limited ATP supply during times of low oxygen [6]. The elimination of a significant number of energy-requiring, function-related processes of a neuron could possibly act to protect it during times when its capacity to generate energy is compromised [7].

Both mammals and insects generally enter a coma state in response to anoxia, characterized by a severe loss of ion homeostasis in brain tissue [8], [9]. In mammals, anoxia rapidly leads to cell death in neural tissue [10]. Insects, however, are able to recover from hours, days and even months without oxygen [8], [11]. The locust *Locusta migratoria* is tolerant of oxygen deprivation, withstanding hours of water submersion and is able to survive 6 hours in an atmosphere of pure nitrogen [12], [13]. The mechanisms of anoxia/hypoxia tolerance in insects are little understood and of potential relevance to the biomedical field [14]. In the locust, both the time to succumb to anoxia, as well as the recovery time from anoxia can be modulated through the activity of highly conserved signaling pathways, including NO/cGMP/PKG [12] and AMP-activated protein kinase, AMPK [15].

AMPK plays a central role in balancing the activities of ATP consuming and producing pathways. The AMP-activated protein kinase is highly sensitive to a small change in the concentration of AMP [16], [17]. AMPK is also localized close to the plasma membrane and its activation is known to reduce both large and background conductance channels inducing membrane depolarization [18]. The location of the AMPK and its sensitivity to minor fluctuations in the AMP:ATP ratio make it an attractive candidate for investigations into the down-regulation of membrane permeability/function in response to energetic stress. AMPK activity has been shown to be necessary and sufficient for changes seen in motor pattern generation in response to anoxic stress in the locust, where it contributes to both the timing and severity of coma-like events [15]. It is currently not known how AMPK contributes to anoxic coma responses in the locust at a cellular level. It is therefore of interest to examine AMPK activation in the context of its role in mediating the effects of anoxic stress on high-performance neurons.

We have investigated the tuning of neural performance following a brief period of anoxia in a well-characterized visual pathway in the locust, the LGMD/DCMD looming motion sensitive circuit. Under normal conditions, the patterning of activity in the LGMD/DCMD circuit in response to approaching objects is relatively fixed for a given target size and approach velocity [19]. The timing and electrophysiological properties of APs elicited by a natural stimulus can be monitored from the large diameter axon of DCMD, allowing detailed investigation of the changes to neural function following anoxic stress. We have tested the hypothesis that the AMPK pathway modifies the cellular properties of the DCMD neuron in a way that mimics the effects of recovery from anoxic stress. We further identify cAMP and HCN as putative cellular elements which may link AMPK activation to the observed changes in cellular physiology.

## Materials and Methods

### Animals

Adult male locusts, *Locusta migratoria*, 3–6 weeks past the final imaginal molt were used for all experiments. Animals were kept in cages of 100–200 aged-matched animals at a cage temperature that varies from 25°C–30°C on a 12 hr∶12 hr light:dark cycle. Animals were fed once daily, and were raised on a diet of wheatgrass and a mixture of bran, milk powder, and yeast.

### Preparation

Locusts were removed from their colony on the day they were to be used and all experiments were performed within 8–10 hours. A semi-intact preparation was used for all electrophysiology experiments, and involved a dorsal dissection of the thorax and abdomen to expose the thoracic nervous system. The precise procedure has been described in detail previously [20]. Once fully exposed, the nervous system was supported on a metal plate to facilitate sharp electrode recording. Care was taken not to damage the tracheal system. For some experiments, the trachea feeding the thoracic ganglia were intentionally cut to induce a hypoxic treatment. The preparation was bathed in room temperature (21°C) standard locust saline containing 147 mM NaCl, 10 mM KCl, 4 mM CaCl_2_, 3 mM NaOH, and 10 mM HEPES buffer (pH = 7.2). Pharmacological agents were dissolved in saline along with a minimum of DMSO as required and applied to the preparation by pipette. All drugs were obtained from Sigma-Aldrich Canada.

### Electrophysiology

Extracellular action potential recordings were made through application of a glass-tipped suction electrode onto the dorsomedial surface of the thoracic nerve cord. Two such electrodes, one anterior and one posterior to the mesothoracic ganglion were used and signals were amplified using an A-M Systems Model 1700 Differential AC amplifier. DCMD activity was clearly distinguishable from other cord activity by its significantly larger amplitude. Intracellular action potential recordings were made with 20–40 MΩ borosilicate glass microelectrodes (WPI) filled with 3M Potassium Acetate, and an A-M Systems Model 1600 Neuroprobe amplifier. DCMD penetrations were made immediately posterior to the mesothoracic ganglion. The preparation was grounded through a silver wire placed in the abdomen. Input resistance at the recording site was calculated by injecting 1 nA of current through a capacitance-compensated recording electrode and measuring the resulting voltage change in the axon. In some experiments, the A-M Systems Differential AC Amplifier connected to the anterior electrode was toggled between recording and stimulation modes, and used in conjunction with an A-M Systems Isolated Pulse Stimulator (Model 2100) to electrically stimulate APs in the DCMD axon. The stimulation strength was set just above the minimum strength required to elicit an AP. All recordings were digitized and saved to computer for subsequent analysis using pClamp software (Molecular Devices).

### Looming visual stimulus

To simulate the looming approach of an object, a video of an expanding black disk on a white background was back-projected onto a screen placed beside the locust preparation using a SHARP XG-C55X digital projector. The image contrast ratio between the black and white regions was 0.92. The video was made using Adobe Flash and contained 3 s of images at 100 frames/sec. The 10 cm×10 cm screen was placed 7 cm perpendicular to the body axis of the animal, with the target disk centered at eye-level. The looming image started at a subtense angle <1° and expanded at an apparent velocity of 1 m/s throughout the 3 s approach to a final subtense angle of 30° at an image diameter of 3.8 cm, which is the size the target would represent at the screen distance of 7 cm from the eye of the animal. The angular subtense (°) of the looming image at any time (t) during the 3 s apparent approach can be expressed as angle (t)  = 2 tan^−1^ [l/v · (t)], where (l) is the target half-size (radius), and (v) is the apparent approach velocity. The l/v for the looming target was 18.5 ms. A sound pulse marker, embedded into the first and final frame of the video, was played through the computer audio output and then digitized. This marker was used to calibrate the object approach to the timing of the animal's response relative to predicted collision of the looming image. Image presentations were separated by at least 2 minutes to reduce the effects of habituation [21]. Between looms, the animal was presented with a pure white image.

### Behavioural assay – anoxic coma

To manipulate AMPK levels, animals were pre-injected with 10 µl of either the AMPK activator metformin (500 mM), the AMPK inhibitor compound C (1 mM), or sham saline 30 minutes prior to a water submersion treatment. Injections were made with a Hamilton syringe inserted between the 3^rd^ and 4^th^ abdominal tergi. Animals were placed in a perforated plastic container and submerged in room temperature water (deionized). Their behaviour was subsequently examined and the time to succumb was measured as the point at which the animal became behaviourally quiescent [12]. This point was clearly identifiable, as it occurred following a brief period of hyperexcitablity. Animals remained underwater for 30 minutes and those used for electrophysiological experiments were allowed to recover in normoxia for 1 hour or longer after return to normoxia. 100% of control animals survived this treatment.

### Behavioural assay – flight responses

Animals were set on a ventrally-fixed tether and flown in a wind tunnel at a wind speed of 3 m/s. Flight time was measured as the total time of active flight, allowing for three terminations in which flight was reinitiated by an abrupt wind stimulus to the head of the animal. Following the time trial, the reliability of steering avoidance behaviour was tested during a flight bout by projecting a looming stimulus at 90° to the head of the animal. Behavioural performance was assessed by simultaneous electromyographic recordings from both the left and right 1^st^ forewing basalar flight muscles M97, which are active during wing depression [22]. Recordings were made by placing an EMG wire through a small hole made in the anterior portion of the thorax and waxing it in place. Muscle activity was amplified using a Model 1700 A-M Systems differential amplifier. Changes in the patterning of motor activity corresponding with the approach of the looming target were scored either as an evasive behaviour if there was a clear and prolonged change in the wingbeat frequencies of both wings or as no change [22].

### Metabolic rate

CO_2_ production was measured by flow-through respirometry and was used as a conservative monitor of metabolic rate. Individual locusts were placed in a 50 cc chamber with a steady stream of air pumped through at 100 mL/min. The air was then dehydrated and sent through a CO_2_ gas analyzer (LoggerPro) which provided a continuous measurement throughout the experiment and was used to calculate a mass-scaled metabolic rate. Animals were allowed to become accustomed to the chamber for 1 hr, and a baseline measurement was taken as the average reading throughout the following 1 hr period. Animals were removed from the chamber and submerged in water for 30 minutes to induce a metabolic coma state. After removal from the water, animals were dried off and promptly returned to the CO_2_ recording chamber. Measurements were made continuously throughout the recovery period. Metabolic rate after recovery from coma was measured 1 hr following the reintroduction to the chamber, and was generated as the average metabolic rate over the subsequent hour.

### Analysis and statistics

The DCMD response to the looming stimulus was recorded, and a pulse embedded in the looming video allowed for the calculation of each individual spike time relative to the predicted collision of the looming disk. Spike timing information was further used to calculate the instantaneous frequency of each DCMD AP relative to the preceding event. A 2D-Gaussian negative exponential smoothing function (20 ms bins; nearest-neighbor) was applied using SigmaPlot on the spike timing data over the 3 s period relating to the looming object approach as well as 200 ms following the predicted time to collision. This was done to facilitate a comparison of the profile of DCMD activity throughout the response to a looming approach between different treatments. Measurement of relative conduction velocity of APs down the DCMD axon was achieved by examining the delay in event time for a given AP between the anterior and posterior electrodes and was used to compare the reliability of transmission down the axon between treatments.

To further assess the importance of different firing rates on conduction reliability, instantaneous frequency of APs during a looming response were binned as <100 Hz, 100–250 Hz, and >250 Hz. The mean relative conduction velocity of APs from each bin was compared at three experimental time points. The first time point was before treatment, the second time point was during treatment (saline or drug), and the third time point was during a saline washout. Statistical analysis for significant differences between two groups was performed using t-tests (P<0.05). Comparison of multiple groups was performed using ANOVA followed by pair-wise analysis corrected for multiple-comparisons (P<0.05).

## Results

### DCMD is a high performance neuron

**Figure 1:**
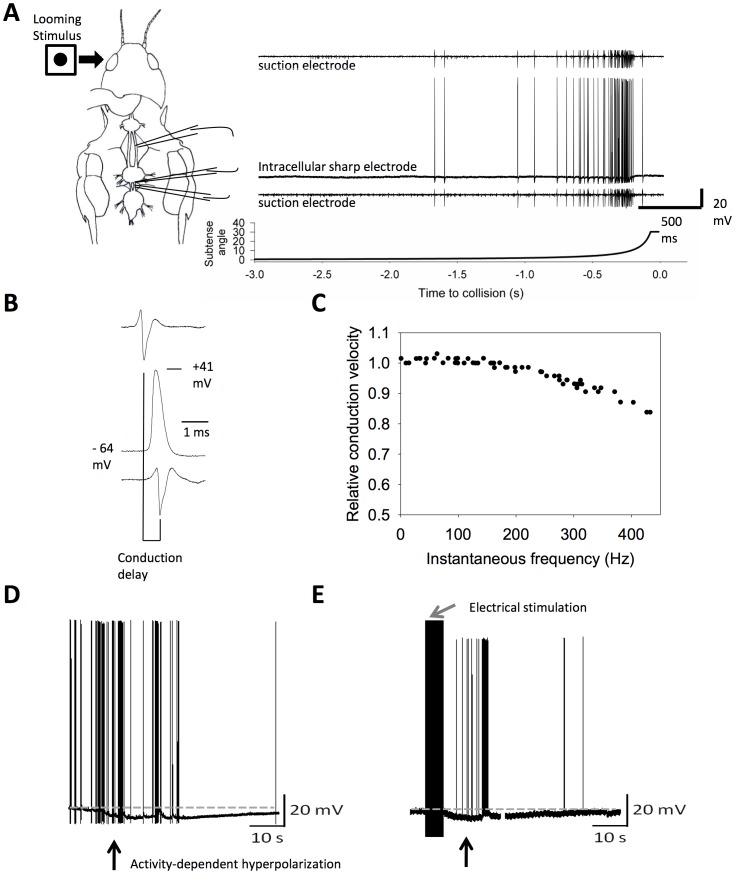
Neural performance can be monitored using the visual looming detector circuit, via the descending contralateral movement detector (DCMD). (A). The timing and electrophysiological properties of APs elicited by a 1 m/s looming stimulus can be recorded from the axon of DCMD. (B). Conduction delay of APs recorded between two extracellular suction electrodes can be used to calculate relative conduction velocity. (C). Quantification of differences in conduction velocity with increasing instantaneous frequency. (D). Activity-dependent hyperpolarization of DCMD (arrow) can be monitored in response to visual stimuli. (E). Electrical stimulation of the axon to evoke 1000 APs (grey arrow) is also sufficient to induce an activity-dependent hyperpolarization (black arrow).

Changes in the properties of the descending contralateral movement detector neuron (DCMD) can be compared during responses to naturalistic looming stimuli (Figure 1A). Responses show target size/speed specific patterning [19], which allows for comparison of spike profile between treatments. The length of the thoracic nerve cord enables measurement of conduction velocities down the axon while intracellularly recording action potentials with large amplitude (≈100 mV) and short duration (<0.5 ms; Figure 1B). Further, this neuron regularly fires trains of action potentials at high frequency (>300 Hz) and at relatively high conduction velocities for unmyelinated axon (3 m/s at 25°C, [23]). Conduction velocities can be sustained even at high frequency (Figure 1C), demonstrating an ability to reliably transmit signals of high intensity. During periods of high activity, either through vigorous visual stimulation (Figure 1D) or by stimulating the axon electrically (Figure 1E), the DCMD axon exhibits an activity-dependent hyperpolarization. APs with prominent afterdepolarizations, as well as increased membrane excitability events such as post-inhibitory rebound are observed under some circumstances [23].

### Neural performance is reduced after recovery from anoxic stress

Anoxia rapidly affects the function of neural tissue in vertebrates and invertebrates alike, with most animals undergoing a period of neuronal depolarization followed by the silencing of electrical activity (coma, [24]). Recovery of neuronal function after return to normoxia is associated with the reestablishment of ion gradients that are disrupted by the anoxic episode [25]. We aimed to examine the extent of change in neuronal processing following recovery from such coma using electrophysiological recordings from the DCMD. Anoxia was induced by submerging the whole animal in room temperature water for 30 minutes, followed by a 1 hour normoxic recovery. Most animals had righted themselves by 30–40 minutes after return to air.

**Figure 2:**
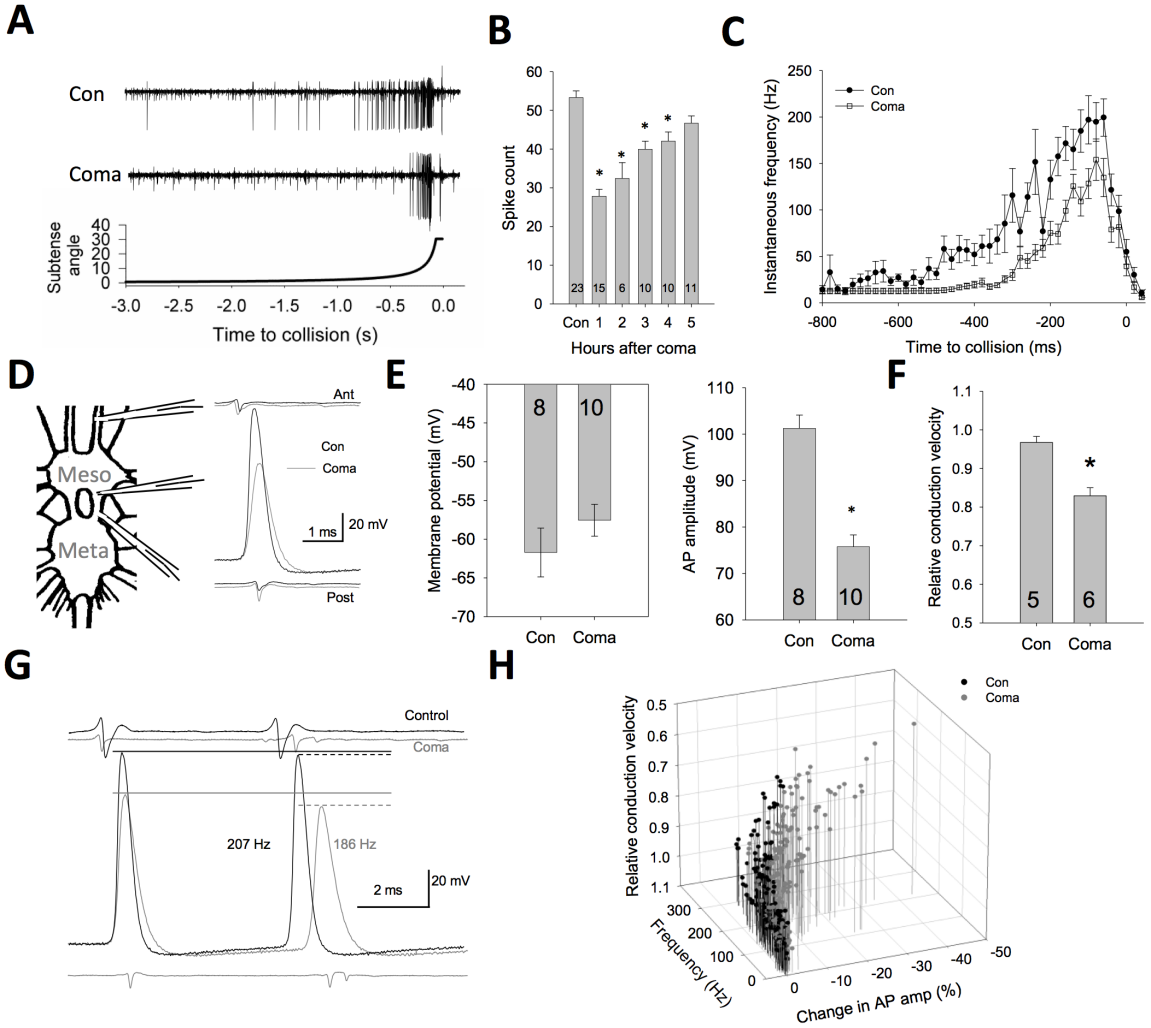
Performance is temporarily reduced after anoxic coma induced by water immersion. (**A**). Raw traces of DCMD activity in response to a looming target stimulus (bottom panel) demonstrate a delay of the response relative to target collision as well as a loss of overall spike activity. (**B**). The reduction in mean spike count is most prominent at 1 hour of recovery, and gradually returns to control levels over a period of 5 hours. (**C**). Peristimulus time histogram of grouped data showing instantaneous frequencies of action potentials during target approach with coma recovered animals having a delayed latency and lower peak activity compared to control animals. (**D**). Illustration of recording arrangement with two extracellular electrodes (ant, anterior; post, posterior) across the mesothoracic ganglion and an intracelullar electrode immediately posterior to the ganglion. Traces are overlayed at the start of the intracellular AP rising phase. (**E**). An examination of AP properties of the DCMD axon recorded from this region compared between control and coma recovered animals reveal a mild resting membrane depolarization and a significant reduction in AP amplitude in coma-recovered animals. (**F**). Coma-recovered animals also show a decrease in the conduction velocity between the extracellular electrodes. (**G**). Frequency-dependent AP amplitude attenuation between events with similar instantaneous frequency from control and coma-recovered animals. APs from coma recovered animals both start with APs of lower amplitude and lose amplitude to a higher degree than controls. (**H**). Group scatter data of individual APs during a looming response plotted to show the relationship between AP instantaneous frequency, AP amplitude loss, and conduction velocity. Coma-recovered animals show a marked instability of both AP amplitude and conduction velocity with increasing instantaneous frequency. Asterisks denote statistical significance (P<0.05). Number of animals per condition is noted within the bars, valued as mean ± SE.

Recordings of DCMD activity from the thoracic nerve cord after 1 hr of recovery showed a strongly reduced mean spiking response to a simulated looming target (One-way ANOVA, P<0.001; Figures 2A,B). At 1 hour of recovery, the decrease in spike activity is broadly distributed throughout the looming target approach, as evidenced by a general decrease in instantaneous frequency of DCMD APs in coma-recovered animals (One way ANOVA, P<0.001; Figure 2C). At longer recovery periods, we found a gradual increase in responsiveness with a return to baseline levels by 5 hours post-anoxia.

Action potentials (APs) recorded intracellularly from the DCMD axon were consistently of lower amplitude following coma recovery compared to time controls (t-test, P<0.001) and also displayed slower conduction down the axon (t-test, P<0.001; Figures 2D–2F). We assessed the effect that increases in firing rate have on amplitude between control and coma experiments, and found that amplitude and conduction speed are reduced to a larger extent following coma (Figures 2G, 2H). The observations show that coma-recovered animals have lowered responsiveness in their processing of visual stimuli and that AP transmission is more susceptible to frequency-dependent changes in conduction velocity.

**Figure 3:**
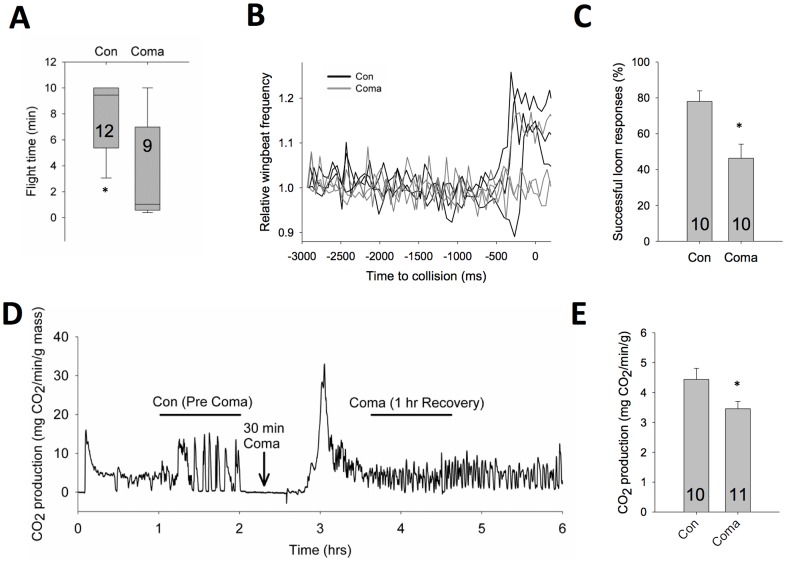
Effect of coma on flight behaviour and basal metabolic rate. (**A**). Coma recovered animals have reduced flight duration in the wind tunnel. Box-plot spans 25^th^ to 75^th^ percentiles, error bars represent 10^th^ to 90^th^ percentile, with solid line as median value. (**B**). Change in wingbeat frequency as measured through EMG activity from thoracic flight muscles during flight in a wind tunnel. Representative data showing deviations from normal wingbeat frequency for three looms trials in each of a control (black) and a coma-recovered animal (grey). (**C**). Control animals respond more frequently to the target approach than coma recovered animals. (**D**). Individual measurement of whole animal metabolic rate using CO2 respirometry prior to anoxic coma and in the recovery period. Basal metabolic rate is initially increased sharply upon return to normoxia, but is then decreased in the following recovery period. Note that prior to coma this animal displayed discontinuous gas exchange characteristic of quiescent animals. Not all locusts showed this pattern and it did not affect the overall CO_2_ measured. (**E**). Metabolic rate is decreased during the post-coma recovery period. Asterisks denote statistical significance (P<0.05).

Given the known role of DCMD in triggering visually-guided escape behaviours [26], we next asked whether the observed change in responsiveness was associated with a change in these behaviours during flight. We inserted EMG wires into the ventral thorax on both the left and right sides into flight muscles (M97; [22]) and flew the animals on a fixed tether in a wind tunnel. Both control and coma-recovered animals showed sustained flight, but the flight durations in coma animals were significantly reduced (t-test, P<0.05; Figure 3A). A looming stimulus was displayed to the flying animal and turning responses were identified by changes in the wingbeat frequency (Figure 3B). Coma animals had significantly reduced responsiveness to the looming stimulus (t-test, P<0.01; Figure 3C). Flight is known to affect the properties of the looming circuit [27], [28], making a direct comparison of the consequences of DCMD activity in the quiescent and flying animal challenging. However, the results from the flight behaviour are consistent with the signalling deficits seen DCMD following coma, and it is suggestive that the reduced DCMD activity may contribute to this behaviourally significant consequence of anoxic coma following recovery.

The observed reductions in neural excitability in the DCMD are likely representative of a nervous system-wide phenomenon. To examine the whole-animal effect of these changes, we looked at the basal metabolic rate of locusts before and after anoxic coma through respirometry (Figure 3D). Following a brief spike in CO_2_ production while the locust is recovering (postanoxic peak; [11]), CO_2_ production is decreased in the period following recovery (t-test, P<0.05; Figure 3E), indicating a lower basal metabolic rate brought on by the anoxic coma.

### Manipulation of AMPK signalling affects DCMD response to anoxic stress

Anoxia results in an energetic deficit in neural tissue. Cellular signalling pathways that regulate energy status in animals are well understood, and a key player is the AMP-activated protein kinase pathway (AMPK; [17]). This signalling pathway responds to an energetic stress by activating catabolic pathways and inhibiting anabolic pathways [16]. While this downregulation of anabolic activity is usually considered within the context of normal cellular metabolism, recent evidence has shown that neuronal excitability may also be targeted [17], [29], [30]. We next tested the hypothesis that activation of the AMPK pathway can mimic the decrease in DCMD activity seen following recovery from anoxic coma.

**Figure 4:**
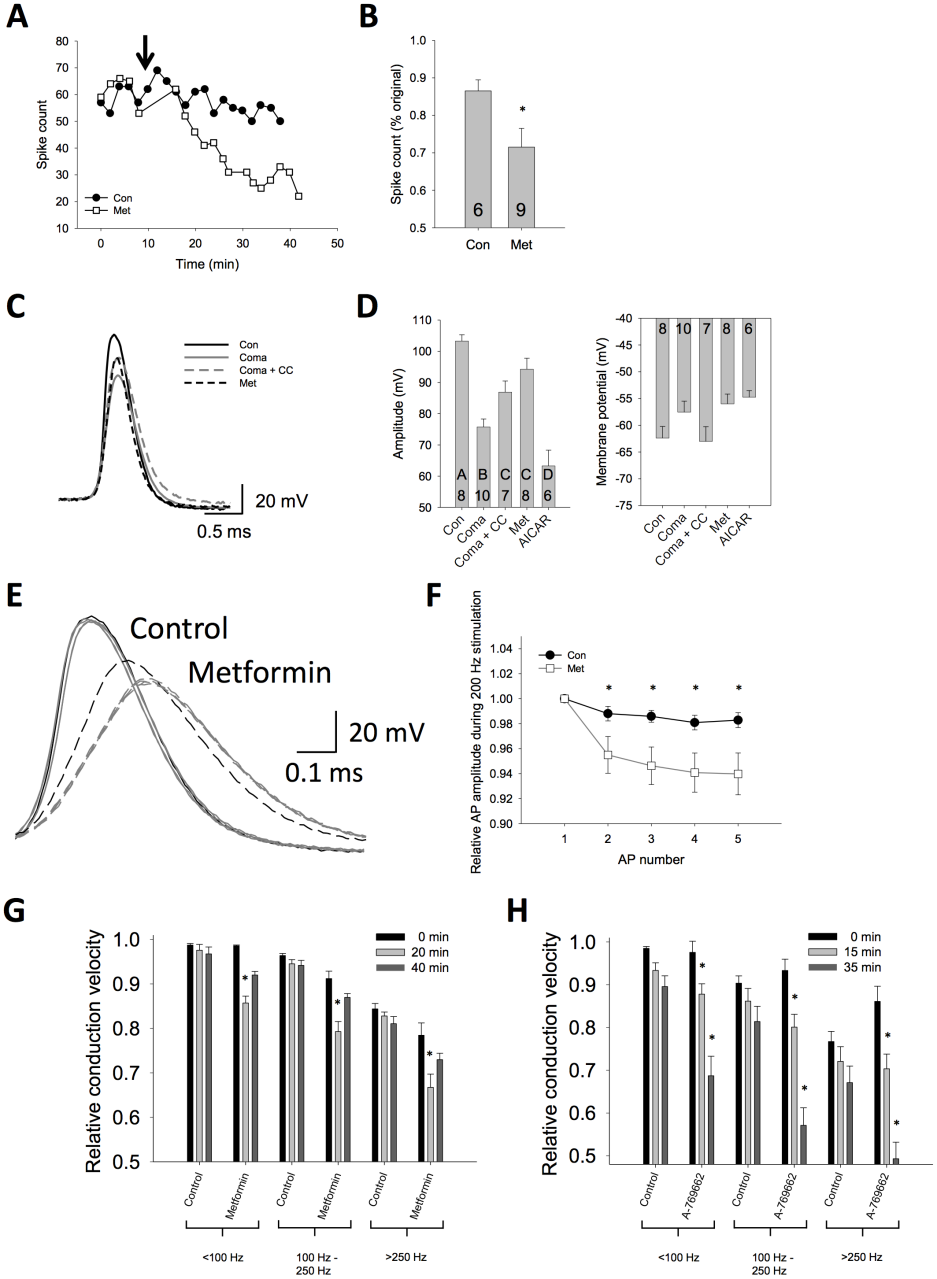
Manipulation of AMPK pathway affects performance in the LGMD/DCMD circuit. (**A**). Metformin application (arrow) results in a decreased responsiveness to repeated looming stimuli. (**B**). Grouped data demonstrating reduced response in metformin (asterisk, P<0.05). (**C**). Axon recordings of DCMD APs have different waveforms after anoxic coma, or following pharmacological manipulation of the AMPK pathway. Metformin (Met, 10 mM) was used to activate AMPK, Compound C (CC, 0.1 mM) was used to inhibit AMPK. (**D**). AMPK inhibition (CC, 0.1 mM) attenuates the effects of anoxic coma on AP amplitude, whereas AMPK activation (Met, 10 mM; AICAR, 1 mM) produces APs with a more coma-like waveform. Statistical significance in amplitude differences denoted by differing letters (P<0.05). These treatments do not show a significant difference in the resting membrane potential. (**E**). Raw trace during repeated 200 Hz stimulation. (**F**). Grouped data showing AP amplitude drops in metformin treated animals but is sustained in control animals (n = 5 Con, n = 9 Met). (**G**). Conduction velocity in the DCMD axon during responses to a looming target. Spikes were binned into groups based on instantaneous frequency (<100 Hz, 100–250 Hz, >250 Hz). Measurements were taken before treatment (0 min), after 20 minutes of either a saline control or metformin treatment (as indicated along the x-axis), and again after a 20 minute saline washout (40 min). Conduction velocity was reduced after 20 min of metformin (asterisk, P<0.05), and recovered after washout. (**H**). Similar effects on conduction velocity are seen with the AMPK activator A-769662, with conduction velocity reduced from pre-treatment levels (0 min) after 15 min of A-769662 and further still after 35 min of A-769662 at all frequency bins (asterisk, P<0.05).

The AMPK activator metformin (Met; 10 mM) was bath applied to preparations and animals were shown a looming target every 2 minutes for 40 minutes. After metformin application, we observed a continual decrease in spike activity per looming response compared to similar preparations with no drug (t-test, P<0.05; Figures 4A, 4B). Further, metformin led to decreased AP amplitude in DCMD axon, while the AMPK inhibitor Compound C (0.1 mM) was shown to recover amplitude in preparations that had been given an anoxic coma treatment (One-way ANOVA with Holm-Sidak pairwise multiple comparisons, P<0.05; Figures 4C, 4D). We also tested the AMPK activator AICAR (1 mM) and found it to have a potent effect on APs resulting in decreased amplitude (Figure 4D). This was associated with increases in conduction delay progressing to a conduction failure in the axon over the course of the experiment (10–45 minutes; data not shown). After bath application of metformin, intracellular recordings showed a reduced ability of the axon to sustain high frequency firing initiated by electrical stimulation. In particular, there was a significant drop in amplitude after the first spike of a bout of 200 Hz electrical stimulation (Two-way repeated measures ANOVA with Holm-Sidak pairwise comparisons, P<0.05; Figures 4E,4F).

Fast axonal conduction is a main feature of the LGMD/DCMD circuit, fitting with its role in initiating escape behaviours. Conduction speeds of 3 m/s are commonly seen at room temperature and can reach 6 m/s at physiologically relevant body temperatures [23]. Such fast conduction is supported by a relatively large axonal diameter (≈15 µm; [31]). Changes in the AP waveform, as seen following AMPK activation, would be expected to affect conduction velocity. To determine the effect of AMPK activation on the propagation of APs down the DCMD axon, we measured DCMD conduction velocity over a stretch of thoracic nerve using two suction electrodes while bath applying metformin (10 mM) to the preparation. By binning the spikes from the looming response according to instantaneous firing frequency (<100 Hz, 100–250 Hz, >250Hz), we found that metformin compromised spike train propagation by slowing conduction velocity of APs down the axon compared to pre-metformin levels or control (Two-way repeated measures ANOVA with Holm-Sidak pairwise multiple comparisons, P<0.001; Con: n = 5, Met: n = 7; Figure 4G). These effects could be washed out by a return to standard saline in the preparation.

To further demonstrate the potential role of AMPK in modulating conduction velocity in the DCMD, we used the AMPK activator A-769662 [32]. Conduction velocity was decreased significantly within 45 minutes in the presence of 1 mM A-769662 compared to control preparations, particularly for APs transmitted at high instantaneous frequencies (>250 Hz, Two-way repeated measures ANOVA with Holm-Sidak pairwise multiple comparisons, P<0.001; Con: n = 10, A-769662: n = 10; Figure 4H). The overall actions of A-769662 on DCMD are similar to that of metformin, strengthening the claim that AMPK activation can mimic the effects of anoxia on aspects of spiking activity and action potential waveforms.

### Role for cAMP and HCN channels in high-performance conduction

**Figure 5:**
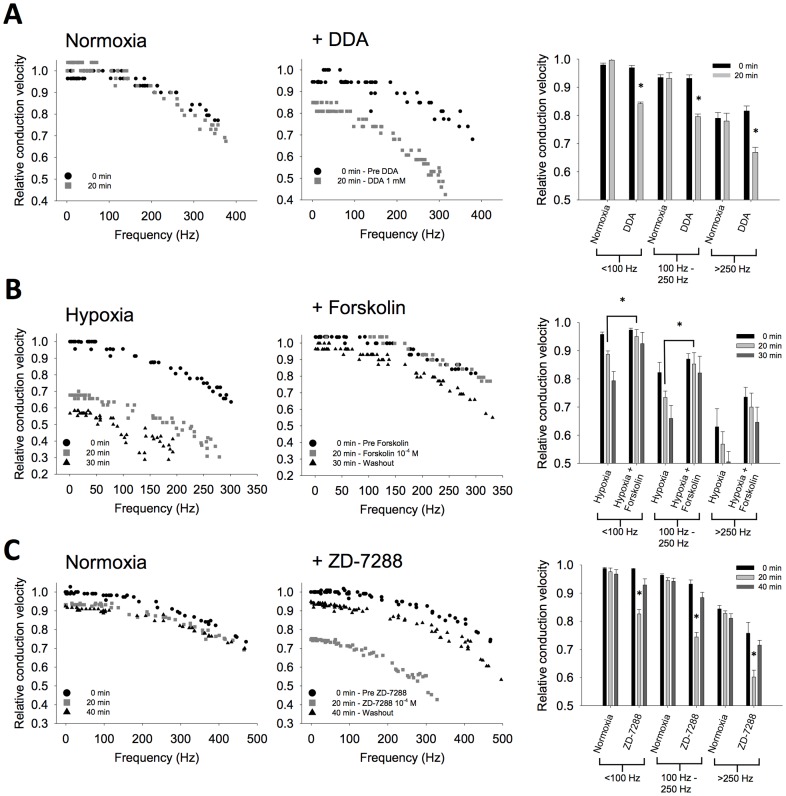
Involvement of cAMP in the DCMD axonal response to a metabolic stress. (**A**). Pharmacological blockade of cAMP activity by bath application of DDA (1 mM) results in an impairment of conduction velocity down the DCMD axon of otherwise healthy animals (Normoxia; P<0.001). Continual DDA bath application led to conduction failure in the majority of preparations, preventing examination of washout. Quantified responses to DDA across firing frequency are shown in the right panel. (**B**). Inducing hypoxia by cutting trachea to the thoracic nervous system reduces conduction velocity in the DCMD axon. The loss of conduction velocity can be mitigated by bath application of the cAMP activator forskolin (100 µM). Right panel displays grouped data showing that forskolin protects AP conduction across different firing rates during hypoxia (P<0.05). (**C**). Reduced conduction velocity in the DCMD axon during responses to a looming target in HCN channel blocker ZD 7288 (100 µM; P<0.001). The effect is reversible and more pronounced during high frequency activity (right panel).

cAMP-dependent 2^nd^ messenger pathways are involved in controlling plasticity in response to changes in the abiotic environment [33]. We wanted to test if manipulation of this pathway might produce similar effects in DCMD to those observed following anoxic coma or AMPK-activation. We examined the effect of blocking cAMP activity on conduction reliability in normoxic, healthy preparations. The preparation was perfused with either normal saline or 1 mM 2′,5′-Dideoxyadenosine (DDA), an inhibitor of adenylyl cyclase that reduces cAMP. DCMD responses to a looming target were recorded, and mean relative conduction velocities of APs were binned by their instantaneous frequency into <100 Hz, 100–250 Hz, and >250 Hz groups. Normoxic control preparations had stable conduction velocities for the duration of the experiment (Figure 5A). In contrast, when 1 mM DDA was applied, relative conduction velocities significantly decreased for all firing frequency bins (<100 Hz, 100–250 Hz, >250 Hz; Two-way repeated measures ANOVA with Holm-Sidak pairwise comparison, Con: n = 5, DDA: n = 5; P<0.001; Figure 5A). Washout of DDA was not measured, as the treatment led to a failure in AP conduction in 80% of trials, which did not recover in the time course of the experiment.

We next created hypoxic locust preparations by severing the tracheal system supplying oxygen to the ventral nerve cord, and animals were bath-applied either normal saline or saline with the adenylyl cyclase activator forskolin (10^−4^ M). Over the length of the 45 minute experiment, we found that hypoxic preparations with normal saline showed a decline in DCMD conduction velocity compared to those with forskolin. We found that forskolin helped sustain conduction velocity for frequency bins of <100 Hz and 100–250 Hz (Two-way repeated measures ANOVA with Holm-Sidak pairwise comparison, Hypoxia: n = 3, Hypoxia+Forskolin: n = 5; P<0.05; Figure 5B), but not >250 Hz (Two-way repeated measures ANOVA with Holm-Sidak pairwise comparison, Hypoxia: n = 3, Hypoxia+Forskolin: n = 5; P = 0.10). This result suggests that increasing cAMP protects against the acute effects of hypoxic stress on conduction velocity in the axon.

Many axons are known to express a variety of voltage-gated ion channels in addition to fast sodium and delayed rectifier potassium channels [34]. HCN channels are known to play a role in facilitating high frequency firing through maintained spike timing and temporal precision [35], [36], and can be gated by cAMP [37]. We used the HCN channel blocker ZD 7288 to determine if a loss of an HCN current could mimic the changes in conduction delay seen following coma as well as during metformin treatment. As found following metformin, 100 µM ZD 7288 increased the conduction delay of APs compared to control during looming responses (Figure 5C). There was a difference in relative conduction velocity in the ZD 7288 treated group comparing pre treatment to drug in all three frequency bins of <100 Hz, 100–250 Hz, as well as >250 Hz (Two-way repeated measures ANOVA with Holm-Sidak pairwise multiple comparisons, Con: n = 5, ZD 7288: n = 5; P<0.001). After washout, conduction velocities in all three bins improved.

### Loss of activity-dependent hyperpolarization by ouabain and metformin

**Figure 6:**
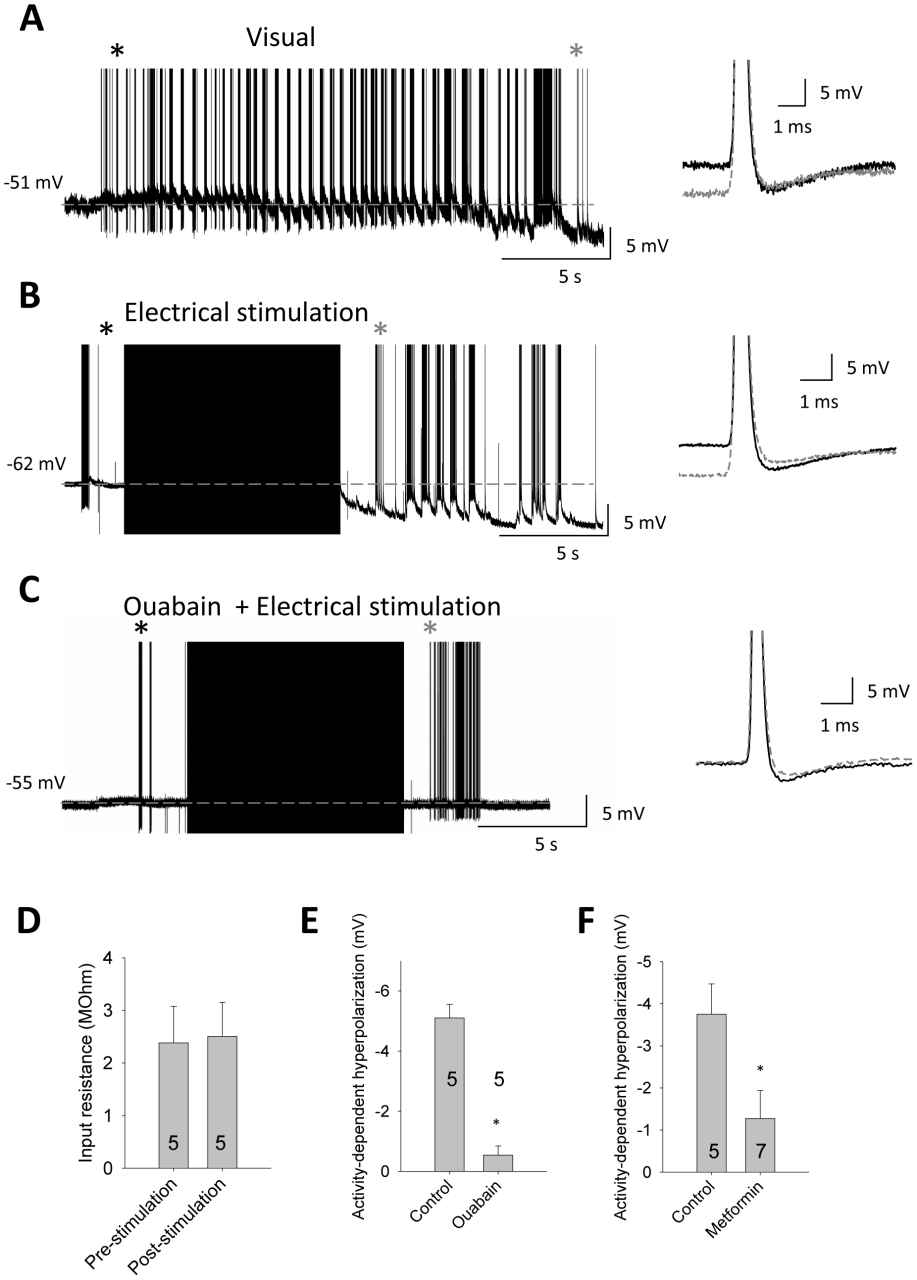
Loss of an activity-dependent hyperpolarization in the DCMD axon following AMPK pathway activation. (**A**). The DCMD axon hyperpolarizes during periods of high frequency activity in response to visual stimulation. The shift in membrane potential occurs with no change in the reversal of the afterpotential (side panel), suggesting that this is an electrogenic effect. Black and grey traces in right-side panel are from time points in raw trace indicated by black and grey asterisks, respectively. (**B**). The membrane potential shows similar hyperpolarizing shifts following electrical stimulation of the axon that generates APs. (**C**). Electrically stimulated activity fails to evoke a hyperpolarization during ouabain treatment (10^−4^ M). (**D**). There is no change in input resistance before the activity-dependent hyperpolarization (pre) compared to during the event (post). (**E**). This hyperpolarization is greatly reduced after ouabain application (10^−4^ M; P<0.05). (**F**). Metformin (10 mM) significantly reduces the hyperpolarization in response to 100 Hz electrical stimulation (P<0.05).

The DCMD axon exhibits an activity-dependent slow hyperpolarization. This event can be initiated either by vigorous visual stimulation (Figure 6A) or by electrical stimulation of the axon to generate a train of APs (Figure 6B). A variety of stimulation periods and stimulation frequencies (>100 Hz) were found to be sufficient to induce the slow hyperpolarization, with repeated trains of shorter duration required in some cases to reduce stimulation failures (data not shown). The hyperpolarization was typically 3–6 mV, and lasted from 10s of seconds up to a minute or more. During the slow hyperpolarization we see no change in the trajectory of the AHP (Figures 6A,6B inset). The electrogenic effect of the Na^+^/K^+^-ATPase pump has been shown in a variety of systems [38], [39] to produce a similar activity-dependent hyperpolarization, and we attempted to manipulate the pump through application of the pump blocker ouabain (10^−4^ M). Pump block with ouabain (10^−4^ M) eliminated the activity-dependent hyperpolarization (Figure 6C). We observe no change in input resistance before compared to during the slow hyperpolarization (pre = 2.4 MΩ±0.7, during = 2.5 MΩ±0.6; paired t-test, P = 0.62; Figure 6D), further suggestive of an electrogenic pump effect. Ouabain was found to significantly reduce the size of the activity-dependent hyperpolarization in response to electrical stimulation (t-test, P<0.05; Figure 6E).

In all of our experiments with anoxia-treated animals we have not observed a strong activity-dependent hyperpolarization in response to looming stimuli, although these animals were not tested using structured electrical stimulation (data not shown). However, these slow hyperpolarizations are significantly reduced in the presence of metformin (t-test, P<0.05; Figure 6F). We suggest that pump activity is reduced by AMPK activation.

### Manipulation of AMPK affects behavioural response to anoxia

**Figure 7:**
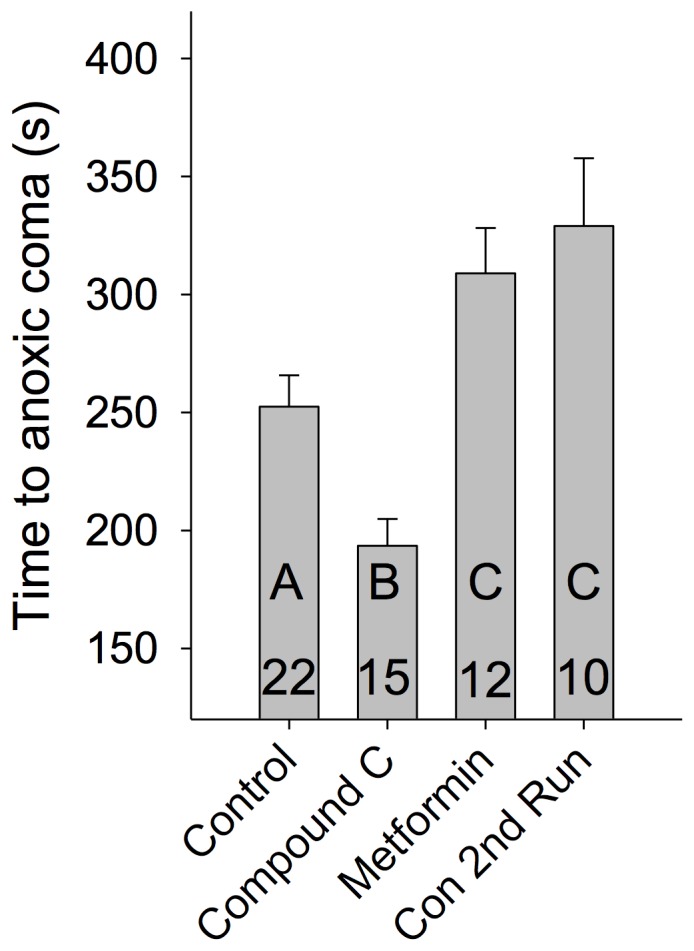
Anoxic preconditioning and AMPK activation is protective against subsequent anoxic events. Animals were submerged in water and the time until they entered a coma state was measured. Preinjection with 10 µL of 1 mM Compound C shortened the time until coma, and metformin preinjection (10 µL, 500 mM) prolonged the animal's ability to remain active in anoxia. A preconditioning effect was found, in that animals that had recovered for one hour from a previous water submersion showed increased time to coma on subsequent anoxia. The preconditioning effect was similar to preinjection with metformin. Statistical significance is denoted by differing letters (P<0.05).

Our results show that following recovery from anoxic coma, or during pharmacological treatment to activate AMPK, the LGMD/DCMD circuit shows a reduced excitability. If these effects represent a general neural phenomenon, then it is possible that the observed changes in neural activity observed following anoxic coma may help to ration energetic resources. We predicted that activation of AMPK would increase the ability of these animals to resist future anoxic events. We examined this by injecting animals with 10 µL of either metformin (500 mM), Compound C (1 mM), or sham saline and subsequently exposed them to a water submersion. The time required to enter metabolic coma was assessed between these groups and compared to control (naive) and preconditioned (1 hour after recovery) animals. Compound C significantly decreased the time to succumb to the water submersion compared to controls, whereas metformin had a protective effect by increasing the time to succumb to a level equal to preconditioned animals (One-way ANOVA with Holm-Sidak pairwise multiple comparisons, P<0.05; Figure 7).

## Discussion

We have shown that excitability of an important visual neuron (DCMD) in the locust exhibits plasticity in response to anoxic stress. These changes can be mimicked through exposure of the nervous system to activators of AMP-activated protein kinase (AMPK), a well described sensor of energetic status in animals. We suggest that the effects of anoxic stress act at least in part through AMPK to lower the energetic demand of neural tissue by reducing excitability. Further, our data support that changes induced by anoxic stress and pharmacological activation of AMPK impart an advantage on the whole animal when faced with subsequent metabolic stress.

It has become increasing clear that neural circuits are tuned to optimize energetic cost and performance [3], [40]. Such optimizations shape circuit design and neuronal branching structure [41], and even the properties of individual action potentials [4], [5]. These energetic features are usually thought of as static in nature, or show plasticity over long time scales resulting from structural changes in brain morphology. Our data demonstrate that the properties of the LGMD/DCMD neural circuit are modifiable by the environment on shorter timescales from minutes to hours, with potential effects on the energetic requirements of this circuit. Given that action potentials account for a significant portion of a neuron's energy budget [3], broadly reducing the number of action potentials generated in response to a given stimuli would be an effective means of reducing energy demand. Indeed, we observed significant reductions in spike counts in the LGMD/DCMD circuit following recovery from coma. While these reductions in DCMD represent only a small component of energetic resources required to process and respond to a visual cue in a freely behaving animal, it may be representative of a general mechanism of reduced excitability in neurons following anoxic coma. The mechanism of reduced activity was not directly addressed here, but given that DCMD follows its presynaptic partner LGMD in a 1∶1 manner [42]–[44], we expect that these changes may be prior to initiation of spike activity in LGMD, rather than uncoupling of the 1∶1 relationship at the LGMD:DCMD synapse. Future work that examines the effects of anoxia on highly energetic upstream components of the visual pathway, such as the photoreceptors and associated optic lobe circuitry would substantiate this claim. We suggest that this reduced spike activity may come at a behavioural cost of reduced likelihood of generating successful escape behaviours associated with this circuit.

High AP conduction velocity is also energetically costly, and accordingly the diameter of axons and leak conductance measures are optimized for energetic cost to maximize firing frequency [45]. High velocity in DCMD is supported by its relatively large axon diameter [31]. Both signalling capacity and energetic cost increase with larger diameter, potentially putting constraints on firing rate by favouring minimally acceptable signal propagation [46]. Inherent in such optimizations is that the limiting level of energetic expenditure is going to be set by the energetic resources of the animal, with tighter constraints on energy-limited animals.

After anoxic coma we saw an altered shape of the DCMD action potential and slowed conduction velocity. These effects on signalling by anoxic stress can be mimicked by targeting AMPK as well the 2^nd^-messenger signalling pathway cAMP. A key cost of action potential signalling arises from the overlap of sodium and potassium conductance [4], [47], [48]. Action potential amplitude is a reasonably good indicator of overall energetic cost, with large APs being more costly [4]. Action potentials are made more efficient by reducing the Na^+^ load by decreasing the time constants for activation and inactivation [4]. A reduced Na^+^ conductance available to support AP propagation could consequently lead to the reduced fidelity seen during high frequency activity. Though not directly tested here, a prediction from our results would be that anoxic stress reduces Na^+^ load. This could occur directly via modulation of the channel kinetics or as a result of increased Na^+^ channel inactivation [5]. The observed decrease in performance following anoxic stress may thus represent a dynamic means of decreasing energy demand in this neural circuit following a severe decrease in energy availability.

While much is known about the role of AMP-activated protein kinase (AMPK) as a sensor of energetic status [16], recent work has focused on the importance of AMPK as an energy sensor in brain tissue [49], [50]. Neurons have relatively small energy reserves, making them particularly susceptible to variations in energy status. AMPK is activated in brain tissue in response to ischemia and hypoxia [51]–[53], glucose starvation [54], as well as during natural states of metabolic depression such as estivation [55].

The role of AMPK in neuroprotection during metabolic stress, however, remains controversial. AMPK activation has been shown to be protective under stress conditions such as cryostorage [56], ischemia [57], chronic hypoxia [58], and glucose deprivation [59]. However, it has also been shown *in vivo* to be associated with increased tissue damage following ischemia [51], and inhibition of AMPK can be neuroprotective in some cases [53], [60].

We show that AMPK activation can reduce responsiveness and excitability in a neural circuit in response to behaviourally relevant stimuli, and that whole animal injection of an AMPK activator is protective for future metabolic stressors. AMPK directly affects excitability in neurons [29] and as well plays a role in LTP [30], highlighting the potential for AMPK to link energetic status to neuronal excitability in a similar way as we propose. Inhibition of the cAMP pathway induces anoxic stress-like changes in DCMD axon, whereas cAMP activation helps sustain conduction reliability during hypoxia. We identify a role for HCN channels in regulating temporal precision and the fidelity of AP signalling. HCN is positively modulated by cAMP in a phosphorylation-independent manner [61], [62], and therefore could act as a downstream axonal target for cAMP. Although we can not directly link AMPK to changes in HCN, the AMPK activator metformin and HCN channel blocker ZD7288 both affect spike train propagation in the DCMD axon in a similar way to anoxic coma by reducing conduction velocity. A signalling pathway in which AMPK's mechanism of action would be to decrease the pool of cytosolic cAMP available to HCN channels, thus reducing HCN activity at rest, is consistent with the data reported here. Changes in conduction velocity occur more substantially at higher AP instantaneous frequencies, and this will consequently have a distorting effect on the temporal structure of the spike train encoded in the brain during its transmission to neurons in the thoracic ganglia. The temporal patterning of APs in DCMD is thought to be important to generate appropriate escape behaviour [63]. Our flight data is consistent with the hypothesis that the observed changes in conduction velocity and firing pattern following recovery from anoxic coma might translate into different behavioural outcomes. This possibility, however, needs to be directly tested in downstream neurons.

HCN channels are known to play a diverse and important role in the excitability of many classes of neurons [62]. HCN helps transmission of spike activity at high frequencies through axon, and decreased axonal HCN lowers conduction fidelity [35], [64], [65]. Such changes are highly activity-dependent, and the relationship of HCN and other axonal currents to changes in membrane potential can be highly non-linear [35]. Further, HCN current is found to be reduced by hypoxia [66], [67], which shifts the activation curve of the current to more hyperpolarized values.

We also see an AMPK-mediated reduction in activity-dependent hyperpolarization in the DCMD axon. Based on the finding that there is no change in the trajectory of the afterpotential, nor any change in the input resistance during this slow hyperpolarization, we suggest that this event is due to the electrogenic effect of the Na^+^/K^+^-ATPase pump. This suggestion is strengthened by the reduction of the activity-dependent hyperpolarization found during treatment with the pump blocker ouabain. Activity-dependent electrogenic effects of the Na^+^/K^+^-ATPase pump can have potent and lasting effects on the properties of neurons [38], [39], [68]. While we have not tested the level of activity-dependent hyperpolarization induced by controlled electrical stimulation in coma recovered animals, we do not observe this in response to looming stimuli following anoxic coma. This preliminary result leads us to predict that controlled electrical stimulation of the DCMD axon would not produce a strong hyperpolarizing response. However, as anoxia-recovered animals also show decreased spiking activity in response to looming targets, the effect of controlled electrical stimulation needs to be tested directly in future work. Hypoxic episodes, such as transient ischemia, can reversibly reduce the membrane-localized fraction of Na^+^/K^+^-ATPase [69]. Indeed, application of the AMPK activator AICAR produces an increase in the extracellular K^+^ concentration in locust ganglion [15], suggesting a reduced Na^+^/K^+^-ATPase activity following AMPK activation. Additionally, metformin-dependent changes to the shape of the action potential may reduce the number of ions per action potential that need to be redistributed, thereby lowering pump demands during periods of activity.

Both HCN and Na^+^/K^+^-ATPase have been shown to participate together in setting the resting potential and membrane excitability, with hyperpolarization by the electrogenic effect of the pump activating HCN [70], [71]. Activity-dependent hyperpolarization driven by the pump helps to prevent Na^+^ inactivation while HCN improves conduction reliability by preventing increases in spike latency related to effects on threshold [35], [64], [65]. It is possible that HCN and Na^+^/K^+^-ATPase are dynamically regulating the excitability of DCMD in a similar manner.

In summary, these results are supportive of the hypothesis that the response to anoxic coma is at least partially under the control of AMPK, a conserved molecular pathway known to be involved in regulating cellular energy status. We identify cAMP as a potential cellular mediator between AMPK and HCN. Together, this would represent a dynamically-regulated means to link the energetic demands of neural signalling with the environmental constraints faced by the whole animal. In interpreting our results, it is important to note that the primarily pharmacological approach we have used does not directly implicate AMPK as necessary and sufficient to produce the observed changes during recovery from anoxic stress. Further, our experiments do not rule out the role of non-neuronal targets mediating some of the drug effects we report. This may be particularly significant for behavioural modifications shown following whole-animal injection. Despite these caveats, this investigation provides a potential mechanism by which neural circuits can modify their cellular function to adaptively limit energetic demand following periods of metabolic stress. Future studies into the contribution made by these AMPK-dependent changes to downstream targets and ultimately behaviour will shed further light on their mechanistic importance.
